# P-selectin-mediated platelet adhesion promotes tumor growth

**DOI:** 10.18632/oncotarget.3164

**Published:** 2015-02-09

**Authors:** Cuiling Qi, Bo Wei, Weijie Zhou, Yang Yang, Bin Li, Simei Guo, Jialin Li, Jie Ye, Jiangchao Li, Qianqian Zhang, Tian Lan, Xiaodong He, Liu Cao, Jia Zhou, Jianguo Geng, Lijing Wang

**Affiliations:** ^1^ Vascular Biology Research Institute, Guangdong Pharmaceutical University, Guangzhou, Guangdong 510006, China; ^2^ Department of Gastrointestinal Surgery, The Third Affiliated Hospital, Sun Yat-sen University, Guangzhou, Guangdong 510630, China; ^3^ Department of Biologic and Materials Sciences, University of Michigan School of Dentistry, Ann Arbor, Michigan 48109, USA; ^4^ Key Laboratory of Medical Cell Biology, China Medical University, Shen Yang City, Liao Ning Province 110001, China; ^5^ Chemical Biology Program, Department of Pharmacology and Toxicology, University of Texas Medical Branch, Galveston, TX 77555, United States

**Keywords:** P-selectin, platelets, tumor growth, αIIbβ3, talin1

## Abstract

Blood platelets foster carcinogenesis. We found that platelets are accumulated in human tumors. P-selectin deficiency and soluble P-selectin abolish platelet deposition within tumors, decreasing secretion of vascular endothelial growth factor and angiogenesis, thereby suppressing tumor growth. Binding of the P-selectin cytoplasmic tail to talin1 triggers the talin1 N-terminal head to interact with the β3 cytoplasmic tail. This activates αIIbβ3 and recruits platelets into tumors. Platelet infiltration into solid tumors occurs through a P-selectin-dependent mechanism.

## INTRODUCTION

Platelets profoundly promote cell transformation, survival and growth by releasing several kinds of growth factors, such as platelet-derived growth factors (PDGFs), fibroblast growth factors (FGFs), vascular endothelial growth factors (VEGFs) and epidermal growth factor (EGFs) [1, 2]. Platelet-derived TGFβ induces an epithelial-mesenchymal-like transition and promotes tumor metastasis [3]. Platelets can aggregate around tumor cells to protect them from clearance by immune-mediated pathways [4]. Platelets can also recruit leukocytes, which secrete pro-inflammatory and pro-thrombogenic cytokines as well as chemokines for tumor angiogenesis, vascular homeostasis, coagulopathy and fibrinolysis [5–7]. Notably, a discontinuous endothelial lining, hyperpermeability and sluggish blood flow are well-documented characteristics of tumor microcirculation [5–7]. Although overwhelming clinical and experimental evidence demonstrates that the depletion of platelets by a variety of mechanisms reduces tumor growth and metastasis [5–7], the therapeutic depletion of platelets is not clinically feasible due to the high risk of potentially fatal hemorrhage. Therapeutic approaches that selectively target platelet interactions with cancer cells within solid tumors without concomitant bleeding complications are thus in urgent need for treating cancers.

The selectin (CD62) family of cell adhesion molecules includes L-selectin (CD62L), E-selectin (CD62E) and P-selectin (CD62P), a pre-synthesized protein stored on the membrane of platelet α-granules and endothelial Weibel-Palade bodies [8–10]. Upon inflammatory or thrombogenic challenges, P-selectin rapidly translocates to the cell-surfaces of endothelial cells and platelets and binds to PSGL-1 (CD162), which mediates leukocyte tethering and rolling on stimulated endothelial cells and the heterotypic aggregation of activated platelets to leukocytes. Platelet glycoprotein GPIbα in the GPI-IX-V complex also acts as a receptor for P-selectin [11]. P-selectin also played an important role in the growth and metastasis of human colorectal carcinoma, attesting to the pathological significance of P-selectin in carcinogenesis [12–15]. However, the mechanism by which P-selectin deficiency attenuates the growth and metastasis of colorectal cancer remains unraveled.

Integrins are a large family of transmembrane heterodimeric adhesion molecules, including 18 α subunits and 8 β subunits. They non-covalently associate to form 24 different integrins expressed on the cell-surface of almost all eukaryotic cells, which are essential for cell-cell and cell-matrix interactions and communications [16–17]. Talin1 contains a high affinity binding site for integrins cytoplasmic tails in the ~50-kDa N-terminal head (THD) and multiple binding sites for actin, vinculin and homotypic dimerization, as well as a second binding site for the integrin cytoplasmic tail in the ~220-kDa rod domain [17]. P-selectin has been shown to act synergistically with cytokines and chemokines to fully activate β_2_-integrins, leading to the firm adhesion of leukocytes to the endothelium during inflammatory responses [18, 19]. Although talin1 binding to the integrin cytoplasmic tail is required for integrin activation, the mechanism by which talin1, abundantly expressed in almost all mammalian cells, is activated via “inside-out” signaling for THD to activate integrins remains unclear.

In this study, we sought to determine the role of P-selectin-mediated platelet adhesion in solid tumors using the Rip1-Tag2 mouse model and xenograft tumor model. Our findings demonstrate that P-selectin deficiency abolishes platelet deposition within solid tumors. Rip1-Tag2 transgenic mice spontaneously develop tumors via multistep progressions, as a consequence of the expression of the SV40 T antigen oncogene in β-cells [20]. Hyperplastic islets begin to appear at the 4–5th week of gestation and angiogenic islets begin to appear at the 6th week of gestation. Some of these angiogenic islets (15–20%) participate in tumors at the 9–10th week [20]. Therefore, the Rip1-Tag2 mouse model was developed to investigate the multistage pathways of carcinoma in a relatively synchronous and predictable progression pattern. Furthermore, we demonstrate that P-selectin activates platelet integrins via the induction of this novel P-sel-CT-talin1-β3 complex, leading to platelet deposition within solid tumors for promoting tumor growth. More importantly, our results support that by targeting this newly identified platelet deposition within solid tumors, soluble P-selectin may afford significant advantages over other therapeutics for treating cancers.

## RESULTS

### Platelets accumulate in human cancers and insulinoma

**Figure 1:**
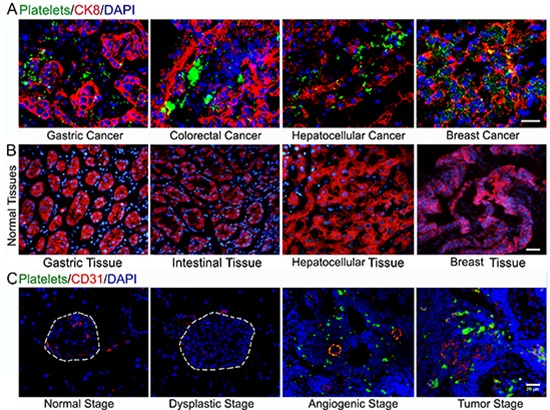
Platelets accumulate in human carcinomas and insulinoma **(A)** Deposition of platelets within human cancers. **(B)** Platelet aggregates were completely absent in the corresponding normal tissues. **(C)** Immunofluorescent staining of GPIbβ-positive platelets, CD31-positive endothelial cells and DAPI-positive cell nuclei in the pancreas islets isolated from control C57BL/6J mice and Rip1-Tag2 mice. Bar = 20 μm.

To test whether platelets infiltrate into solid tumors, we have examined platelet deposition in several types of human cancer specimens using immunofluorescent staining of βIIb for platelets, cytokeratin 8 (CK8) for tumor cells and DAPI for nuclear DNA. Surprisingly, we found prominent accumulation of platelet aggregates around tumor cells in every specimen of human colorectal cancer (*n* = 13), gastric cancer (*n* = 12), hepatocellular cancer (*n* = 3) and breast cancer (*n* = 5; Figure 1A). Of note, platelet aggregates are completely absent in the corresponding normal tissues (Figure 1B).

To further assess platelet deposition in solid tumors, we labeled murine platelets with DyLight 488-conjugated anti-GPIbβ antibody (Ab) *in vivo* and examined platelet localization in Rip1-Tag2 mice, which spontaneously develop pancreatic islet insulinoma [20]. Pancreatic islets were analyzed by immunofluorescent staining vascular endothelial cells with CD31 and staining nuclear DNA with DAPI. We observed overt platelet aggregates within the angiogenic islet and tumor, while platelet aggregation was lost in normal, hyperplastic and dysplastic islets (Figure 1C). Furthermore, H&E staining was performed to determine the histologic appearance in the Rip1-Tag2 mice tumor progression (Supplemental Figure 1).

### P-selectin-mediated platelet accumulation accelerates insulinoma growth

**Figure 2:**
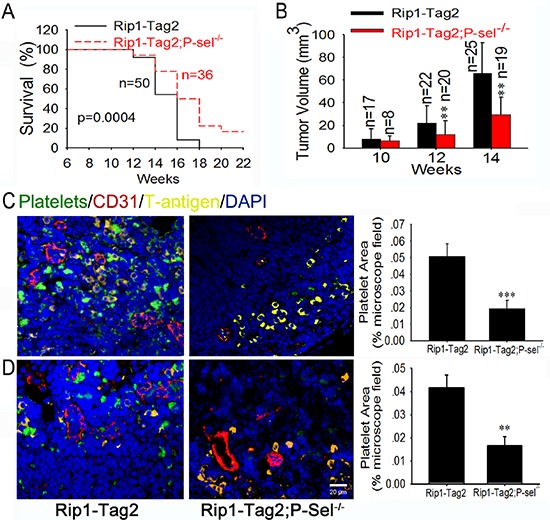
P-selectin mediates platelet deposition and promotes insulinoma growth **(A** and **B)** Survival rates (A) and tumor volumes (B) of Rip1-Tag2 and Rip1-Tag2;P-sel^−/−^ mice. Tumor volume was calculated as the sum of individual tumor volumes of every mouse in the group. **(C** and **D)** Immunofluorescent images of insulinomas from Rip1-Tag2 and Rip1-Tag2;P-sel^−/−^ mice. The results represent at least five sections per animal from a minimum of three mice per group (C and D). *p < 0.05; **p < 0.01; ***p < 0.001. Bar = 20 μm.

To explore the mechanism of platelet accumulation in tumor, we examined the pathological significance of P-selectin within insulinomas. The genetic deletion of P-selectin increased the survival rate of Rip1-Tag2; P-sel^−/−^ mice (Figure 2A) in comparison with Rip1-Tag2 mice. Rip1-Tag2;P-sel^−/−^ mice also displayed smaller tumor volumes when compared to their Rip1-Tag2 counterparts, especially at the age of 12–14 weeks (Figure 2B). Furthermore, no gastrointestinal bleeding, determined by fecal hemo-occult testing, was observed in any Rip1-Tag2, P-sel^−/−^ or Rip1-Tag2;P-sel^−/−^ mice (data not shown). In analogous to P-sel^−/−^ mice [21], Rip1-Tag2;P-sel^−/−^ mice displayed moderately prolonged bleeding (Supplemental Figure 2).

To further test whether platelets accumulate in insulinomas, we labeled murine platelets with DyLight 488-conjugated anti-GPIbβ Ab *in vivo* and detected platelet deposition in the insulinomas of Rip1-Tag2 mice. Pancreatic islets were analyzed by immunofluorescent staining of CD31 for vascular endothelial cells, SV40 large T-antigen (T-antigen) for tumor cells and DAPI for nuclear DNA. We observed overt platelet aggregates around tumor cells within the insulinomas (Figure 2C). Only platelet aggregates were sufficiently large to be detected (data not shown). Platelet aggregates were also visible within blood vessels (Figure 2C and Supplemental Figure 3). When Rip1-Tag2 mice were crossed with P-sel^−/−^ mice, designated as Rip1-Tag2;P-sel^−/−^ mice, the infiltration of platelet aggregates into insulinoma was almost undetectable (Figure 2C). Alternatively, we isolated mouse platelets from transgenic mice expressing green fluorescent protein (GFP) and intravenously injected them into Rip1-Tag2 mice (Figure 2D). In addition to these discoveries within blood vessels (Figures 2C, 2D and Supplemental Figure 3), platelets adhered to T-antigen-positive tumor cells within insulinomas (Figures 2C and 2D). Furthermore, vascular endothelial cells were also independently verified by immunofluorescent staining with an anti-von Willebrand factor (vWF) Ab (Supplemental Figure 4).

To support our findings in the murine model of insulinoma, we tested whether P-selectin could enhance platelet infiltration and accelerate tumor growth in other tumor models. Following subcutaneous inoculation of B16 cells, P-sel^−/−^ mice manifested mitigated platelet recruitment (Supplemental Figure 5A), prolonged overall survival (Supplemental Figure 5B) along with reduced tumor volume (Supplemental Figure 5C) and size (Supplemental Figure 5D) in comparison with C57BL/6J mice.

### P-selectin-Fc suppresses insulinoma growth by abolishing platelet accumulation within tumors

**Figure 3:**
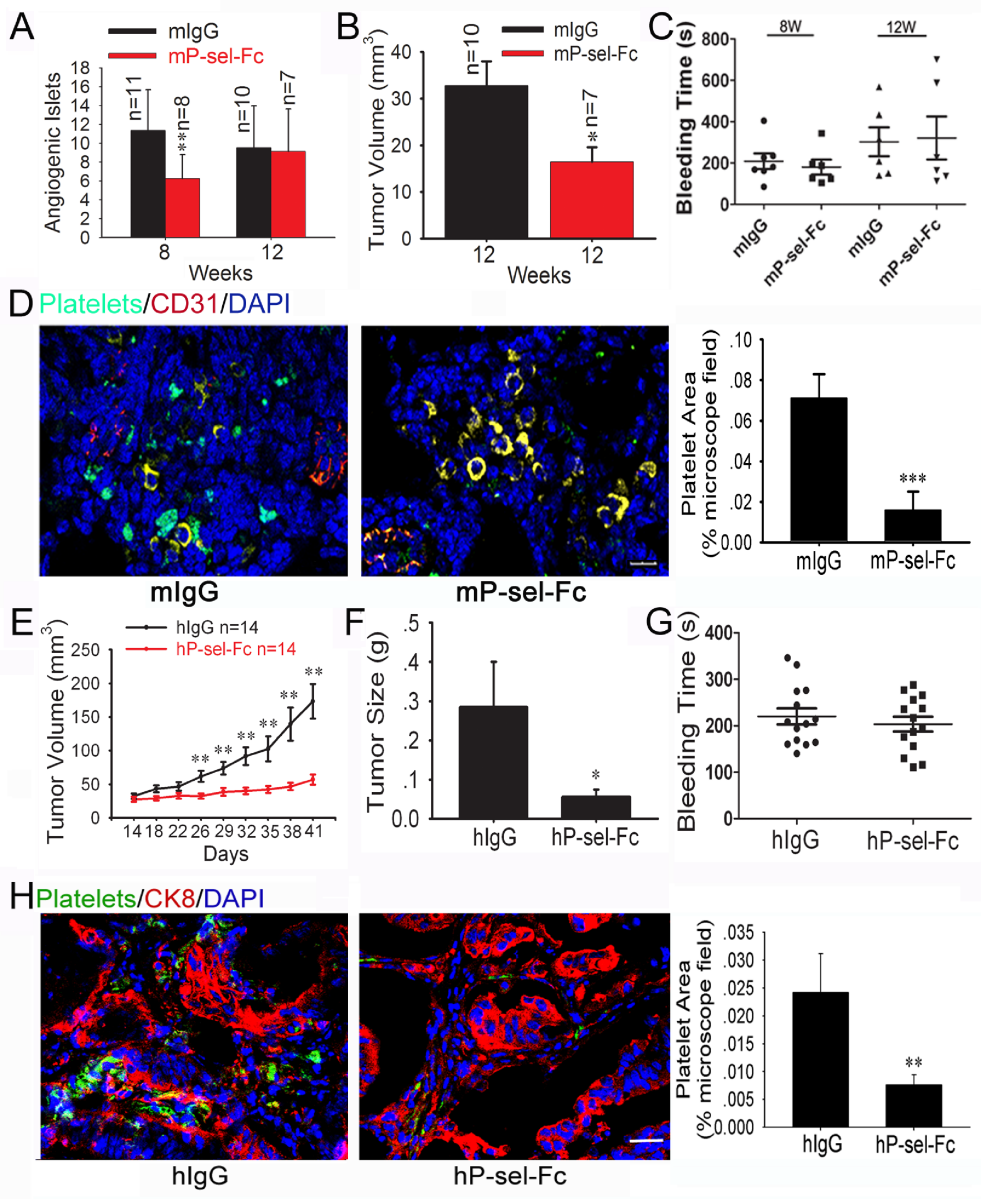
Soluble P-selectin inhibits tumor growth and abolishes platelet recruitment in Rip1-Tag2 mice and xenografted human colorectal cancer **(A** and **B)** Effects of mP-sel-Fc on insulinoma growth and angiogenesis in Rip1-Tag2 mice. Following intravenous administration of mIgG or mP-sel-Fc through the tail veins of Rip-Tag2 mice, tumor volume (A) and angiogenic islet number (B) were assessed. **(C)** mP-sel-Fc did not affect the bleeding time of Rip1-Tag2 mice. **(D)** Imunofluorescent images of GFP-expressing platelets, T-antigen-positive tumor cells (yellow), CD31-positive endothelial cells (red) and DAPI-positive cell nuclei (blue) in pancreas islet insulinomas were examined and showed that mP-sel-Fc abolished the infiltration of platelets into insulinoma. **(E** and **F)** The colorectal cancer-bearing mice were treated intravenously with hIgG or hP-sel-Fc. The tumor volume and weigh were calculated. **(G)** The effect of hP-sel-Fc on bleeding time of mice. **(H)** The accumulation of platelets in xenografted colorectal cancer significantly decreased. *p < 0.05; **p < 0.01. Bar = 20 μm.

Because P-selectin-mediated platelet accumulation accelerates insulinoma growth, we recombined mP-sel-Fc and hP-sel-Fc [22] to treat the 5-weeks-old or 9 weeks-old Rip1-Tag2 mice for three weeks and xenografted human colorectal cancers. We found that the intravenous administration of mP-sel-Fc, but not mouse IgG, suppressed angiogenic islets in 8-weeks-old Rip1-Tag2 mice and volume in 12-weeks-old Rip1-Tag2 mice (Figures 3A and 3B), but did not inhibit the angiogenic islets in 12-weeks-old Rip1-Tag2 mice (Figure 3A). At 5–8 weeks, 4 Rip1-Tag2 mice (*n* = 11) bore insulinomas following mIgG treatment while none of them (*n* = 8) had any insulinomas following mP-sel-Fc treatment (data not shown). Furthermore, the mP-sel-Fc abolished platelet adhesion in the insulinomas of Rip1-Tag2 mice (Figure 3D).

To further investigate the effect of hP-sel-Fc on tumor growth, we established mouse xenograft tumor models using fresh specimens of human colorectal cancer. Interestingly, compared to hIgG, hP-sel-Fc potently inhibited growth of xenografted human colorectal cancers (Figures 3E and 3F) and abolished infiltration of mouse platelets into xenografted tumors (Figure 3H). Notably, compared to hIgG, hP-sel-Fc had no detectable effects on bleeding times in athymic nude mice xenografted with the tissue specimens of human colorectal cancers (Figure 3G). Furthermore, gastrointestinal bleeding, determined by fecal hemo-occult testing, was not observed in any mice following mP-sel-Fc and hP-sel-Fc treatment (data not shown). Thus, our results not only demonstrate the clinical relevance of our experimental findings in mice, but also support the potential efficacy of soluble recombinant human P-selectin for treating human cancers via the neutralization of platelet deposition and inhibition of tumor growth. We believe that the potential complication of soluble P-selectin-induced bleeding or hemorrhage during cancer treatment warrants further clinical investigation, although the risk for soluble P-selectin-induced bleeding or hemorrhage appears to be low and unlikely (Figures 3C and 3G).

### Platelets promote insulinoma growth

**Figure 4:**
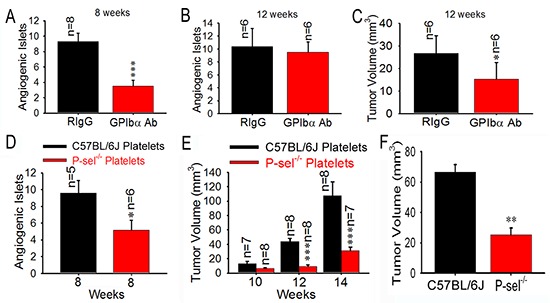
Role of platelet P-selectin in platelet accumulation and tumor growth **(A–C)** Effects of platelet depletion on angiogenic islets (A and B) and tumor growth (C). **(D** and **E)** Effects of infused platelets on angiogenic islets (D) and tumor growth (E). Wild-type or P-sel^−/−^ platelets were intravenously administered to Rip1-Tag2;P-sel^−/−^ mice. Angiogenic islets and tumor volumes were measured as described above. **(F)** Bone marrow cells from wild-type and P-sel^−/−^ mice were intravenously injected into the Rip1-Tag2;P-sel^−/−^ mice and the tumor volumes were calculated. *p < 0.05; **p < 0.01; ***p < 0.001.

Because platelets aggregate in insulinoma, they could play an important role in insulinoma growth. Next, we examined the effects of the temporary depletion of circulating platelets on insulinoma growth. An intraperitoneal injection of GPIbα Ab, but not its isotype-matched rat IgG (RIgG), drastically reduced blood platelet counts on average by > 90% (~0.8 × 10^6^/μL), causing gastrointestinal bleeding in all tested mice (data not shown). To investigate whether anticancer efficacy of GPIbα Ab is associated with the hypoxia, the immunohistochemical staining of hypoxia-inducible factor-1 alpha (HIF1-α) was carried out, and we found that the tumor tissues of Rip1-Tag2 mice treated with GPIbα Ab and RIgG showed high levels of HIF-1α immunoreactivity and there was no difference between GPIbα Ab and RIgG group (Supplemental Figure 6). Nevertheless, platelet depletion for 4 weeks significantly reduced angiogenic islets at 8 week (Figure 4A), but not at 12 week (Figure 4B), and decreased tumor volume 2-fold in Rip1-Tag2 mice at 12 weeks compared to the controls (Figure 4C).

### Platelet P-selectin, but not endothelial cell P-selectin, mediates platelets aggregation, thereby promoting insulinoma growth

P-selectin is expressed on activated platelets and vascular endothelial cells [8]. To eliminate the role of endothelial cell P-selectin and elucidate the effect of platelet P-selectin, we isolated platelets from C57BL/6J and P-sel^−/−^ mice, respectively. We found that platelets from C57BL/6J mice, but not those from P-sel^−/−^ mice, rescued angiogenic islets (Figure 4D) and tumor volume (Figure 4E) in Rip1-Tag2;P-sel^−/−^ mice. In addition, bone marrow from C57BL/6J mice, but not from P-sel^−/−^mice, significantly enhanced tumor volume in Rip1-Tag2;P-sel^−/−^ mice (Figure 4F).

### Engagement of P-selectin activates αIIbβ3 through talin1

**Figure 5:**
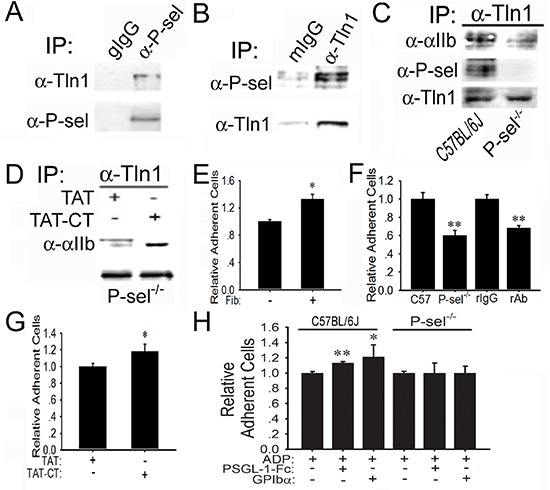
Induction of P-sel-CT-talin1-β3 complex in platelets **(A** and **B)** P-selectin binds to talin1. Lysates of wild-type platelets isolated from C57BL/6J mice were incubated with goat preimmune IgG (gIgG) or the anti-P-selectin Ab (α-P-sel; A) and mIgG or the anti-talin1 mAb (α-Tln1; B). After extensive washing, the immunoprecipitated proteins were immunoblotted for talin1 or P-selectin, respectively. **(C)** P-selectin induces talin1 binding to αIIb. The lysates of wild-type and P-sel^−/−^ platelets were incubated with the anti-talin1 mAb. After extensive washing, they were immunoblotted for αIIb, P-selectin and talin1. **(D)** P-sel-CT augments talin1 binding to αIIbβ3. The lysates of P-sel^−/−^ platelets were incubated with TAT or TAT-CT and the proteins immunoprecipitated by the anti-talin1 mAb were immunoblotted for αIIb and talin1. **(E–H)** P-selectin enhances platelet adhesion to fibrinogen. Adhesion of isolated wild-type mouse platelets labeled with BCECF to mouse fibrinogen (Fib) (E), in the absence or presence of isotype-matched irrelevant rat IgG2b and the rat anti-GPIIb/IIIa mAb, was performed (F). Additionally, P-sel^−/−^ platelets were preincubated with TAT and TCT-CT (G) or PSGL-1-Fc and GPIbα in the presence of ADP (H). *p < 0.05; **p < 0.01.

To understand why P-selectin blockade could abolish integrin-mediated platelet adhesion to tumor cells within solid tumors (Figures 1A and 1C, Figures 2C and 2D), we tested whether endogenous P-selectin might interact with talin1 in platelets. The anti-P-selectin Ab, but not goat preimmune IgG (gIgG), co-immunoprecipitated talin1 in the lysates of platelets isolated from C57BL/6J mice (Figure 5A). The anti-talin1 monoclonal Ab (mAb), but not mIgG, co-immunoprecipitated P-selectin in the platelet lysates (Figure 5B). To demonstrate the significance of P-selectin in triggering talin1 binding to αIIbβ3, we compared wild-type platelets from C57BL/6J mice and platelets isolated from P-sel^−/−^ mice. Just as predicted, endogenous talin1 from P-sel^−/−^ platelets bound poorly to αIIb compared to its counterpart from wild-type platelets (Figure 5C). Notably, talin1 co-immunoprecipitated P-selectin evidently in wild-type platelets, but not P-sel^−/−^ platelets. To examine whether P-sel-CT would be sufficient to mediate talin1 binding to αIIbβ3, we generated the peptide of cell-permeable P-sel-CT (TAT-CT) and its control peptide (TAT), using the exact strategy previously described [22]. Indeed, TAT-CT, but not TAT, increased co-immunoprecipitation of endogenous talin1 to αIIb in the lysates of P-sel^−/−^ platelets (Figure 5D).

Functionally, compared to the wells without immobilized mouse fibrinogen, platelets isolated from C57BL/6J mice adhered abundantly to immobilized fibrinogen (Fib; Figure 5E), which was inhibited by the neutralizing anti-αIIbβ3 mAb, but not its isotype-matched irrelevant rat IgG (rIgG) (Figure 5F). P-sel^−/−^platelets adhered poorly to fibrinogen compared with wild-type platelets. Preincubation of P-sel^−/−^platelets with TAT-CT, but not TAT, significantly increased adhesion of P-sel^−/−^platelets to fibrinogen (Figure 5G). Compared to adenosine 5′-diphosphate (ADP) alone, preincubation with ADP plus PSGL-1-Fc or GPIbα further increased adhesion of wild-type platelets, but not P-sel^−/−^ platelets, to fibrinogen (Figure 5H).

### Adherent platelets secrete VEGF for induction of angiogenesis

**Figure 6:**
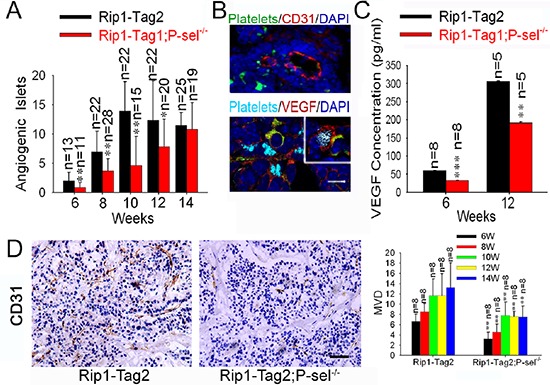
Adherent platelets secrete VEGF, and accelerate angiogenesis **(A)** Effects of P-selectin on tumor-induced angiogenesis. Angiogenic islets were determined in Rip1-Tag2 and Rip1-Tag2;P-sel^−/−^ mice. **(B)** Adherent platelets release VEGF. Immunofluorescent staining of pancreatic islets in insulinoma for platelets (GPIbβ), endothelial cells (CD31) or VEGF. Insert, the insulinomas isolated from Rip1-Tag2 mice were immunofluorescently stained for VEGF, αIIb for platelets, T-antigen for tumor cells and DAPI for nuclear DNAs. **(C)** The total amounts of VEGF in insulinomas. The insulinomas isolated from Rip1-Tag2 and Rip1-Tag2;P-sel^−/−^ mice at 6 and 12 weeks were lysed and the levels of VEGF were determined. **(D)** Microvessel density (MVD) of insulinoma. *p < 0.05; **p < 0.01. Bar = 20 μm.

To explore how recruited platelets enhance tumor growth, we evaluated tumor-induced angiogenesis in Rip1-Tag2 mice and Rip1-Tag2;P-sel^−/−^ mice and found that Rip1-Tag2;P-sel^−/−^ mice had significantly fewer angiogenic islets at the ages of 6–12 weeks (Figure 6A). As platelets are a rich source of VEGF [1, 2], a key factor for induction of angiogenesis in neoplastic islets [23, 24], we examined the expression and localization of VEGF within insulinomas. VEGF was found to be highly expressed in the area of tumor cells expressing T-antigen, where platelet aggregates were apparent (Figure 6B, lower panel). VEGF was also found on the periphery of platelet aggregates adherent to tumor matrix (Figure 6B, insert of lower panel). We next determined the levels of VEGF in the tissue lysates of pancreatic islet insulinomas isolated from Rip1-Tag2 and Rip1-Tag2;P-sel^−/−^ mice. As expected, the amounts of VEGF from the insulinomas of Rip1-Tag2 mice were significantly higher than those from Rip1-Tag2;P-sel^−/−^ mice at both 6 and 12 weeks (Figure 6C). Consistently, P-selectin deficiency reduced microvascular density (MVD) as determined by CD31 staining for vascular endothelial cells (Figure 6D).

## DISCUSSION

Extensive clinical and experimental evidence has demonstrated that platelets play an important role in tumor growth and metastasis. Although it has been reported that the depletion of platelets reduces tumor growth and metastasis [5–7], depletion of platelets has no clinical application value due to the high risk of potentially fatal hemorrhage. Therefore, it is imperative to identify an agent, capable of targeting platelet interactions with cancer cells within solid tumors while lacking of concomitant bleeding complications, to treat cancers. In this study, we have shown that platelets are actively infiltrated to solid tumors, such as insulinoma and malignant melanoma, through a P-selectin-dependent mechanism, where they secrete VEGF, and undoubtedly many other growth factors, thereby leading to angiogenesis and tumor growth. Mechanistically, direct binding of P-selectin cytoplasmic tail to talin1 triggers THD to interact with β3-CT to activate αIIbβ3 and make platelets infiltrate into solid tumors.

During this study, we unexpectedly found that platelet deposition is evident in every examined human carcinoma tissue (Figure 1A), which is fully consistent with previous reports indicating that P-selectin mediates platelet adhesion to several human cancers and human cancer-derived cell lines [10]. Intriguingly, these results suggest a general mechanism for P-selectin-mediated platelet recruitment into solid tumors. To support the speculation, Rip1-Tag2;P-sel^−/−^ mice and a xenograft model of malignant melanoma were established, and P-selectin absence inhibited tumor growth by abolishing the accumulation of platelets in solid tumors (Figure 2 and Supplemental Figure S4). Thus, our findings indicate that P-selectin mediates platelet deposition within insulinoma, which is reminiscent of previous findings that P-selectin mediates arterial and venous thromboembolism [25]. Our previous results [26] vividly recapitulate the experimental findings of reduced hematogenous metastasis in P-sel^−/−^ mice, which have platelets that fail to respond to thrombin [27], as thrombin acts as a potent agonist for the induction of P-selectin cell-surface expression in platelets [8, 9]. Taken together, our results from two different experimental settings indicate that P-selectin potentiates tumor growth likely by mediating platelet adhesion to tumor cells.

To further prove this concept, we treated Rip1-Tag2 mice and colorectal cancer-bearing mice with P-sel-Fc and found that P-sel-Fc significantly inhibited tumor growth by abolishing platelets aggregation (Figure 3). Therefore, both the endogenous and exogenous deletion of P-selectin inhibits platelet accumulation in solid tumors and tumor growth. Thus, we believe that disrupting P-selectin-mediated platelet recruitment into solid tumors warrants clinical investigation in human patients with carcinomas. Moreover, the genetic deletion of P-selectin only mildly prolongs bleeding time [21], and the intravenous administration of soluble P-selectin does not result in detectable gastrointestinal bleeding (data not shown) and does not prolong bleeding time either (Figures 3C and 3G).

Because P-selectin is expressed on the stimulated endothelial cells and activated platelets [10], exploring whether only platelet P-selectin plays an important role in tumor growth is important. We found that platelet P-selectin, but not endothelial P-selectin, plays a critical role in solid tumor growth via platelet “rescue” experiments and bone marrow transplantation (Figure 4). Thus, our findings reveal a previously unrecognized function of platelet P-selectin in mediating platelet adhesion during the pathogenesis of tumor growth. These results are fully consistent with previous findings on the fundamental role of platelets in carcinogenesis using antibody-induced thrombocytopenia, the thrombin inhibitor hirudin, a GPIIb/IIIa antagonist, and Nf-E2^−/−^ mice, which have almost no circulating platelets [27–31].

Although the functional importance of endothelial P-selectin for mediating leukocyte rolling adhesion in inflammation is well characterized and fully appreciated [8–10], the physiological and pathological roles of platelet P-selectin in the processes of hemostasis, thrombosis, wound healing and tumorigenesis are less well understood. We have elucidated that the engagement of platelet P-selectin induces the cytoplasmic tail of P-selectin to bind to talin1 and consequently bind the N-terminal head domain of talin1 to the β3 cytoplasmic tail in order to activate αIIbβ3, thus depositing platelets within solid tumors (Figure 5). Analogous to leukocyte PSGL-1 signaling [22], our newly identified platelet P-selectin signaling pathway acts synergistically with extracellular stimuli, such as ADP, for the optimal activation of integrins and maximal adhesion of platelets (Figure 5H). This novel mechanism of “inside-out” signaling for activating platelet integrins biochemically explains why P-selectin abrogation abolishes platelet accumulation within solid tumors (Figures 1–3 and Supplemental Figures S1 and S3). Our discovery of P-selectin-mediated platelet integrin activation may also explain how P-selectin mediates homotypic platelet aggregation *in vitro* [32, 33] and accelerates arterial and venous thromboembolism *in vivo* [9].

Taken together, in this study we have unexpectedly discovered that platelet deposition is prominent in each and every human carcinoma tissue examined and that recombinant soluble human P-selectin prevents platelet infiltration and consequently inhibits the growth of orthotopically xenografted human colorectal cancer. Intriguingly, recombinant soluble human P-selectin, in analogous to its treatment of arterial and venous thrombosis [9, 33], fails to severely prolong bleeding time and does not cause detectable gastrointestinal bleeding. Thus, we conclude that soluble P-selectin may offer new perspectives for preventing P-selectin-mediated platelet deposition within solid tumors, and targeting this newly identified platelet-cancer cell interaction potentially represents a novel effective therapeutic approach to human cancer treatment.

## MATERIALS AND METHODS

### Ethics statement

Human samples were obtained with informed consent and the project was approved from the Third Affiliated Hospital Ethics Committee of Sun Yat-sen University. All animal experiments were conducted according to relevant national and international guidelines. And all the protocols were approved by the Medical Research Animal Ethics Committee of Guangdong Pharmaceutical University. When tumor volume exceeded 2 cm^3^, mice were euthanized by cervical dislocation.

### Human samples

Cancer patients provided informed consent before tumor specimens were obtained for immunofluorescent staining. The samples were obtained when the patients received surgical treatment and fixed in 4% paraformaldehyde and embedded in optimum cutting temperature (OCT) compound. Sections (6 μm) were obtained for immunofluorescent staining. The samples included normal tissues, gastric cancer (*n* = 12), colorectal cancer (*n* = 13), hepatocellular cancer (*n* = 3) and breast cancer (*n* = 5). Tissues were collected from the Third Affiliated Hospital, Sun Yat-sen University, Guangzhou, China. And this project was approved by the Medical Research Human Ethics Committee of Guangdong Pharmaceutical University and the Third Affiliated Hospital of Sun Yat-sen University.

### Mice

Rip1-Tag2 mice were obtained from NCI (National Cancer Institute, Washington, DC, USA), and their phenotypes were identified as previously described [20]. P-selectin knockout (P-sel^−/−^) mice were purchased from the Jackson lab. Male Rip1-Tag2 mice were mated with female P-sel^−/−^ mice, and their male progeny carried the Rip1-Tag2 transgene. Animals were housed and used in experiments in accordance with institutional guidelines. And the animal protocol was approved by the Medical Research Animal Ethics Committee of Guangdong Pharmaceutical University.

### Blood sampling and platelet preparation

Blood was obtained from P-sel^−/−^, GFP or wild-type mice and collected in Eppendorf tubes containing acid-citrate-dextrose (ACD, 38 mmol/L citric acid, 75 mmol/L trisodium citrate, and 100 mmol/L dextrose, 1/10 volume). The platelet-rich plasma (PRP) was prepared by a 2-step centrifugation (275 g for 14 minutes and 400 g for 5 minutes). The resulting PRP was filtered to isolated platelets through a Sepharose 2B (Sigma, St Louis, MO, USA) column equilibrated with PIPES buffer (25 mmol/L PIPES, 137 mmol/L NaCl, 4 mmol/L KCl, and 0.1% dextrose, pH 7.0). Platelets were counted using a blood cell counting chamber in a 100 × field.

### Immunofluorescent staining

Immunofluorescent staining was carried out using antibodies against CD31 (Santa Cruz Biotechnology, Santa Cruz, CA, USA), SV40 large T-antigen (Millipore, Billerica, MA, USA), CD41 (integrin GPIIb subunit) and vWF (both from Abcam, Cambridge, CB, UK), VEGF (Abcam), cytokeratin 8 (Santa Cruz Biotechnology) and insulin (Dako Cytomation, Carpinteria, CA, USA). Nuclei were also counterstained with 4′-6-Diamidino-2-phenylindole (DAPI). DyLight 488-conjugated anti-GPIbβ Ab (Emfret Analytics, Würzburg, Eibelstadt, Germany) was used to label platelets *in vivo* according to the manufacturer's protocol. Alternatively, GFP-expressing platelets (4 × 10^7^/mouse) isolated from GFP transgenic mice were injected through the tail veins. The platelets were quantified using an image analysis program IPP 6.0 (Image Pro-Plus, version 6.0, Media Cybernetics) in a 400 × field and evaluated by two experimenters.

### Immunohistochemical staining

For immunohistochemical staining, the sections were incubated using anti-CD31 (Santa Cruz Biotechnology) primary antibodies overnight at 4°C. The next day, the HRP-conjugated secondary antibody was added to the tissues and stained with DAB. The microvessel density was assessed by counting the number of CD31^+^ vessels in a 200 × field [34]. The data were independently collected by two experimenters and evaluated using a double-blind protocol.

### Murine model of spontaneous insulinoma

Rip1-Tag2 and Rip1-Tag2;P-sel^−/−^ mice were characterized as previously described [35]. Angiogenic islets and tumors in Rip1-Tag2 and Rip1-Tag2;P-sel^−/−^ mice were quantified as previously described [36]. The VEGF levels in the lysates of the pancreatic islet cells isolated from Rip1-Tag2 and Rip1-Tag2;P-sel^−/−^ mice were determined by ELISA (R&D Systems, Minneapolis, MN, USA).

### Xenograft model of malignant melanoma

All animal experiments were carried out on 6- to 8-week-old female C57BL/6J mice and female P-sel^−/−^ mice. Mouse malignant melanoma B16-F10 (B16) cells (1 × 10^5^/mouse) were subcutaneously (s.c.) injected into the lower flanks of the mice. The length and width of tumors were measured with a vernier caliper, and the tumor volume was calculated using the following formula: length × width^2^ × 0.52 [37].

### Platelet depletion and “rescue” experiments

For the platelet depletion experiments, GPIbα (CD42b) rat polyclonal Ab or its isotype-matched rat preimmune IgG (both from Emfret Analytics) was intravenously administered to Rip1-Tag2 mice (5 weeks old or 9 weeks old; 2 μg/gram of mouse body weight, once every five days for three weeks). Three weeks later, angiogenic islets and tumors in the 8-weeeks-old and 12-weeks-old Rip1-Tag2 mice were quantified as previously described [20]. For the “rescue” experiments, platelets (4 × 10^7^/mouse) isolated from C57BL/6J and P-sel^−/−^ mice [38] were intravenously administratered to Rip1-Tag2;P-sel^−/−^ mice (9 weeks old; three times a weeks for three weeks).

### Tumor models and P-selectin-Fc treatments

Rip1-Tag2 mice (5 weeks old or 9 weeks old) were intraperitoneally treated with isotype-matched irrelevant mouse IgG (mIgG) or recombinant mouse P-selectin-Fc (mP-sel-Fc) (0.5 mg/mouse, twice per week for three weeks). Fresh specimens (2 mm^3^) of human colorectal cancer were orthotopically transplanted into athymic nude mice (6 weeks old) via axillary incisions [39, 40]. Two weeks later, they were intravenously treated with isotype-matched irrelevant human IgG (hIgG) or recombinant human P-selectin-Fc (hP-sel-Fc) [41] (0.5 mg/mouse, twice per week for three weeks).

### Bleeding time

The mouse tail bleeding time was determined as previously described [42]. The mice were intraperitoneally anesthetized prior to tail transsection. The distal 3 mm of the mouse tail was cut off, and the tail was immediately immersed in 37°C normal saline. The bleeding time was recorded and sustained until blood stopped flowing from the tip of the tail without restarting within 30 seconds. Fecal occult blood test was utilized to detect the gastrointestinal bleeding.

### Bone marrow transplantation

The whole bodies of 7 to 8-weeks-old Rip1-Tag2; P-sel^−/−^ mice were irradiated (9 cGy) in two split doses 3 to 4 hours apart, from a cesium source. After Rip1-Tag2; P-sel^−/−^ mice were irradiated, the recipient mice were injected with 5 × 10^6^ bone marrow cells fromP-sel^−/−^ mice and C57BL/6J mice. Rip1-Tag2;P-sel^−/−^ mice were euthanized at the age of 14 weeks, and the tumor volumes were calculated.

### Co-immunoprecipitation

The co-immunoprecipitation experiments were carried out as described previously [43] using an anti-P-selectin Ab (Santa Cruz Biotechnology), an anti-talin1 mAb (Sigma-Aldrich) and an anti-βIIb mAb (Novus Biologicals, Littleton, CO, USA).

### Platelet adhesion assay

Mouse platelets were isolated and labeled with BCECF (Invitrogen, Carlsbad, CA, USA) [40]. Mouse fibrinogen (Fib; Sigma-Aldrich) was added into 96-well tissue culture plates and a platelet adhesion assay was performed as described [44]. For the antibody inhibition experiments, wild-type platelets were pre-incubated with isotype-matched irrelevant rat IgG2_b_ and rat anti-αIIbβ3 monoclonal antibody (mAb, emfret Analytics; both at 10 g/mL) for 20 min at 37°C prior to transferring to Fib-coated wells. In addition, P-sel^−/−^ platelets were preincubated with the peptide of cell-permeable P-sel-CT (TAT-CT), its control peptide (TAT) and GPIbα (all at 10 μg/mL), in the absence or presence of 100 μM ADP (Sigma-Aldrich), for 20 min at 37°C.

### Statistical analysis

Statistical significance was determined with a one-way ANOVA followed by Bonferroni's post-hoc test for multiple group comparisons or Student's *t*-test for two group comparisons. For all tests, *p* < 0.05 or < 0.01 was considered statistically significant or very significant.

## SUPPLEMENTAL FIGURES


