# Supplementary material for: P-selectin-mediated platelet adhesion promotes tumor growth
Source: Oncotarget. 2015 Feb 9;6(9):6584–96. doi: 10.18632/oncotarget.3164 (PMC4466636; doi:10.18632/oncotarget.3164)
Supplement: Supplementary file 1 [file oncotarget-06-6584-s001.pdf]

SUPPLEMENTAL FIGURES

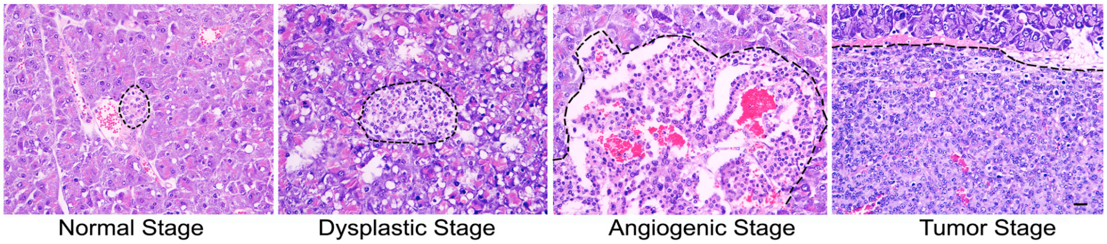

Supplemental Figure S1: The histologic appearance in the tumor progression of Rip1-Tag2 mice. Rip1-Tag2 mice have a multi-step progression to tumors. To determine the histologic appearance in the tumor progression, H&E staining was performed and the image was taken. Bar = 20 µm.

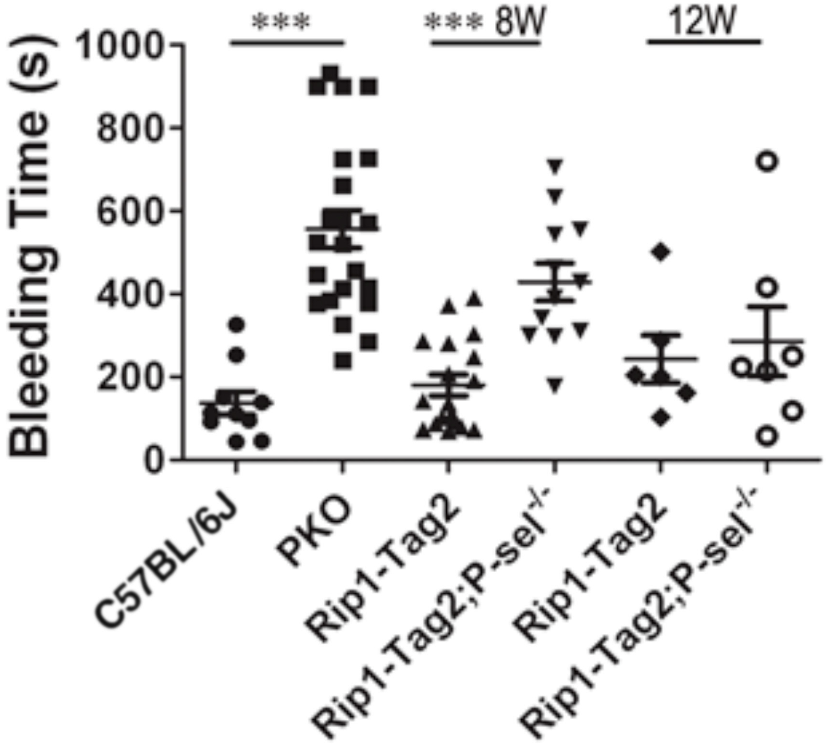

Supplemental Figure S2: Determination of bleeding times. The bleeding time was determined for C57BL/6J, P-sel$^{-/-}$, Rip1-Tag2 and Rip1-Tag2;P-sel$^{-/-}$ mice. ***$p < 0.001$.

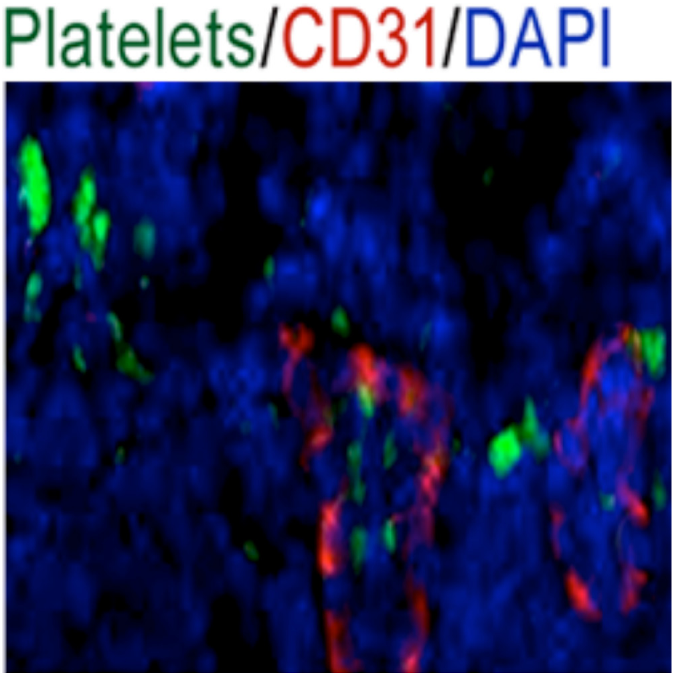

Supplemental Figure S3: Platelets accumulate both within blood vessels and out of blood vessels. Immunofluorescent staining of GPIbβ-positive platelets, CD31-positive endothelial cells and DAPI-positive cell nuclei in the pancreas insulinoma isolated from Rip1-Tag2 mice.

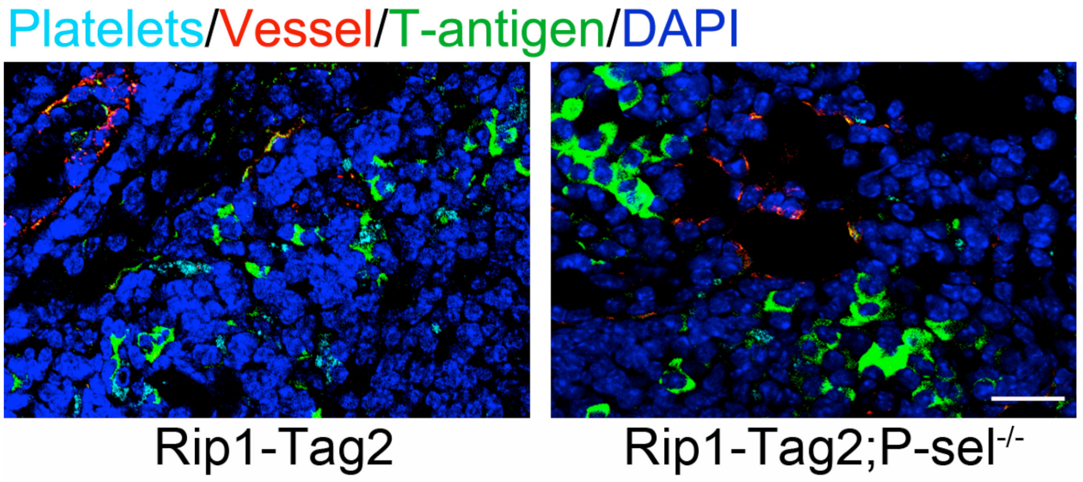

Supplemental Figure S4: vWF Ab immunofluorescent staining of vascular endothelial cells. The sections of pancreas islets were observed following intravenous administration of the DyLight 488-conjugated anti-GPIbβ Ab for platelets and immunofluorescently stained with Abs against vWF for vascular endothelial cells and T-antigen for tumor cells followed by DAPI counterstaining for cell nuclei. Bar = 20 µm.

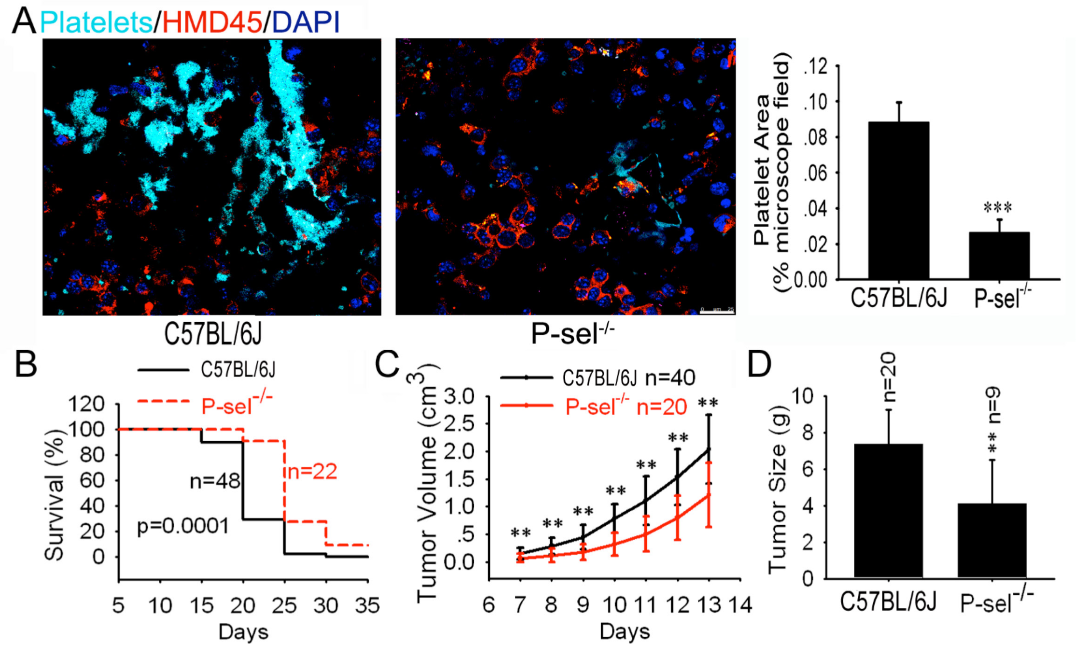

Supplemental Figure S5: P-selectin recruits platelets and accelerates growth of malignant melanoma. (A–D) Measurements of adherent platelets (A) survival rates (B) tumor volumes (C) and sizes (D). The melanoma sections were observed following intravenous administration of the DyLight 448- conjugated anti-GPIbβ Ab and immunofluorescent staining of HMD45 and DAPI. ***p* < 0.01; ****p* < 0.001. Bar = 20 μm.

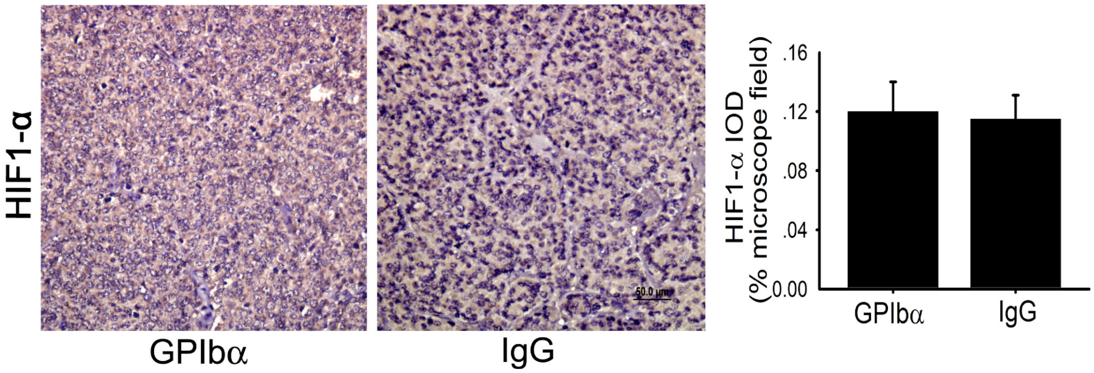

Supplemental Figure S6: GPIbα did not affect expression levels of HIF1-α in tumor tissues. The sections of pancreas islets from Rip1-Tag2 mice treated with GPIbα Abs and control IgG were immunohistochemically stained with Ab against HIF1-α. There was not significant difference between the GPIbα Abs group and IgG group. Bar = 50 μm.
